# Contamination of blood pressure cuffs by methicillin-resistant *Staphylococcus aureus* and preventive measures

**DOI:** 10.1007/s11845-013-0961-7

**Published:** 2013-05-03

**Authors:** M. Matsuo, S. Oie, H. Furukawa

**Affiliations:** Department of Pharmacy, Yamaguchi University Hospital, 1-1-1 Minamikogushi, Ube, 755-8505 Japan

**Keywords:** Blood pressure cuff, Contamination, Methicillin-resistant *Staphylococcus aureus*, MRSA

## Abstract

**Background:**

Although blood pressure cuffs are commonly used and shared in medical facilities, their routine disinfection is performed infrequently.

**Aims:**

We investigated the contamination of blood pressure cuffs by methicillin-resistant *Staphylococcus aureus* (MRSA).

**Methods:**

The MRSA level on the inner side (the surface in contact with patients’ skin) of blood pressure cuffs used in the wards and outpatient clinics of a university hospital (733 beds) was determined using the gauze and swab wiping methods.

**Results:**

Using the gauze wiping method (*n* = 35), the MRSA contamination rate was 31.4 %, and the MRSA contamination level was 1,702.6 ± 9,996.1 (0–58, 320) colony-forming units (cfu)/cuff. No MRSA was detected on blood pressure cuffs after washing (*n* = 30) or wiping with 80 vol% ethanol (*n* = 18).

**Conclusions:**

Blood pressure cuffs are frequently contaminated by MRSA.

## Introduction

Although blood pressure cuffs are commonly used and shared in medical facilities, their routine disinfection is performed infrequently. There have been a few studies of blood pressure cuff contamination by methicillin-resistant *Staphylococcus aureus* (MRSA), but in each study only a qualitative survey of MRSA was performed and the MRSA level was not described [1–3]. In addition, there are few studies on the method for maintaining the cleanliness of blood pressure cuffs [4]. Therefore, we investigated MRSA contamination of blood pressure cuffs using the swab wiping method commonly employed for the detection of contaminants [1, 3–5] and the sterile gauze wiping method [6]. Methods for maintaining the cleanliness of blood pressure cuffs were also evaluated.

## Materials and methods

### Quantification of MRSA on surfaces

The MRSA level on the inner side (the surface in contact with patients’ skin) of blood pressure cuffs used in the wards and outpatient clinics of a university hospital (733 beds) was determined. The investigation period was from September 2009 to January 2010.

Using the swab method, the entire surface of the inner side of the blood pressure cuff was wiped using swabs moistened with sterile physiological saline. The swab samples were placed in 1.0 mL of nutrient broth (Nissui Pharmaceutical Co., Tokyo), stirred for 1 min, and ultrasonicated at 36 kHz for 5 min. Each broth sample was diluted 10^−1^, 10^−2^, 10^−3^, and 10^−4^ fold in sterile saline; 0.2 mL of undiluted sample and of each dilution was placed onto trypticase soy agar and salt egg yolk agar (Nissui Pharmaceutical Co.) and spread with a glass hockey stick. Plates were incubated at 35 °C for 2 days. Yellow colonies on the plates with pearl ring formation in the surrounding medium were examined by Gram-staining, morphological examination, the coagulase test (Staphylo La Seiken, Denka Seiken Co., Tokyo), and an Api Staph kit (Analytab Products, Plain View, New York, USA) to determine whether they were *S. aureus*. Staphylo La Seiken is based on the agglutination method and is composed of a latex suspension that coats human fibrinogen and rabbit IgG. The methicillin sensitivity of cultured *S. aureus* was determined using MRSA screening agar containing 6 mg/mL of oxacillin (Nippon Becton–Dickinson Co., Japan).

Sterile gauze (15 × 10 cm) moistened with sterile saline was also used for wiping blood pressure cuffs and then placed in a 100-mL bottle containing 40 mL of nutrient broth (Nissui Pharmaceutical Co.). The bottle was shaken for 30 min in a recipro-shaker (Taiyo Co., Tokyo; frequency, 250 min^−1^; amplitude, 50 mm) and ultrasonicated (Sine Sonic 100, Ikemoto Rikagaku Co., Tokyo) at 36 kHz for 5 min [7]. Each sample was diluted 10^1^-, 10^2^-, and 10^3^-fold in sterile saline; two aliquots (0.2 mL each) of each dilution and of an undiluted sample were plated on two salt egg yolk agar plates. In addition, the remaining saline sample (ca. 49 mL) in the bottle was filtered through a 0.22-mm membrane filter (Nippon Becton–Dickinson Co.; diameter, 5 cm), which was placed on salt egg yolk agar plates and incubated for 48 h at 35 °C [8]. The methods for determining whether colonies were S. *aureus* and the methicillin sensitivity of cultured *S. aureus* were similar to those used for the swab method. When the membrane filtration technique revealed 50 or fewer colony-forming units (cfu) of *S. aureus*, the results obtained with this technique were used as *S. aureus* counts on the cuff surfaces. When the membrane filtration technique revealed more than about 50 cfu (in instances where *S. aureus* could not be counted), *S. aureus* counts on the cuff surfaces were calculated from the diluted inocula on the egg yolk agar. When the membrane filtration technique or the inoculation onto egg yolk agar technique showed 1–9 cfu of *S. aureus*, the methicillin sensitivity of all these colonies was determined. When either of these techniques showed 10 or more cfu of *S. aureus*, ten colonies were randomly selected and their methicillin sensitivity was determined. The MRSA or methicillin-sensitive *S. aureus* count per surface was estimated from the ratio of methicillin-resistant to methicillin-sensitive colonies.

### Evaluation of methods to maintain cleanliness

The MRSA contamination level on a total of 30 blood pressure cuffs after outsourced washing was determined using the gauze wiping method. In addition, to evaluate the effects of wiping with ethanol for disinfection, the inner side of 18 blood pressure cuffs in use was randomly divided into halves, and one-half was wiped with 80 vol% ethanol and left for 30 min. The contamination level was determined using the gauze wiping method (the MRSA contamination level in the other half was used as the control).

### Statistical analysis

The mean MRSA detection levels were compared by the Wilcoxon *U* test between swab and gauze wiping method.

## Results

Table 1 shows MRSA contamination of blood pressure cuffs evaluated using the swab and gauze wiping methods. The MRSA detection rate was 17.2 % using the swab wiping method and 31.4 % using the gauze wiping method. The MRSA contamination level was 1.9 ± 8.8 cfu/cuff using the swab wiping method and 1,702.6 ± 9,996.1 cfu/cuff using the gauze wiping method, which was significantly higher using the latter (*p* < 0.01).

**Table 1:** Contamination of blood pressure cuffs by methicillin-resistant *Staphylococcus aureus* (MRSA) using the swab and gauze wiping method

Detection method	MRSA contamination rate (%) (no. of samples contaminated/no. of samples examined)	Mean ± standard deviation (range) of MRSA contamination level (cfu/cuff)^a^
Swab wiping	17.2 (10/58)	1.9 ± 8.8 (0–65)
Gauze wiping	31.4 (11/35)	1,702.6 ± 9,996.1 (0–58, 320)

^a^The colony-forming units (cfu)/inner side of blood pressure cuff (surface in contact with patients’ skin)

None of the blood pressure cuffs (*n* = 30) after washing or after wiping with 80 vol% ethanol (*n* = 18) was contaminated with MRSA. However, in the controls without disinfection, the MRSA contamination rate was 22.2 % and the contamination level was 9.6 ± 36.5 (1–160) cfu/cuff by the gauze wiping method.

## Discussion

The swab method is widely used as a simple means to detect the contamination of instruments and environments due to its straightforwardness. However, the results of this study showed that the swab method is considerably inferior to the gauze wiping method for MRSA detection. Therefore, it is possible that MRSA contamination surveys underestimate contamination. In the future, the gauze wiping method should also be used in surveys of the contamination of instruments and environments.

Blood pressure cuffs are shared instruments, but at present may not be washed or disinfected regularly. Regular washing or disinfection of blood pressure cuffs was performed in only one ward of the dermatological department among 14 wards and outpatient clinics of the university hospital investigated in this study. In this ward of the dermatological department, blood pressure cuffs were washed at 7-day intervals. The cuffs from this ward had a lower level of MRSA contamination.

At the Japanese hospital in the fiscal year 2009, the rate of MRSA transmission was 0.3 nosocomial MRSA cases per 100 hospital admissions. This investigation of contamination status using the gauze wiping method showed MRSA on 11 (31.4 %) of 35 blood pressure cuffs examined. The MRSA detection rate was therefore high. One sample showed 5.8 × 10^5^ cfu of MRSA. In a previous investigation of MRSA contamination of blood pressure cuffs, Walker et al. [1] reported a contamination rate of 8 % using the swab method, and de Gialluly et al. [2] reported a contamination rate of 4.4 % using the contact plate method. The contamination rate in this study was far higher than that found in those studies. It is possible that blood pressure cuffs contaminated by MRSA become infection sources, and the maintenance of the cleanliness of cuffs is therefore important.

Methods for maintaining the cleanliness of cuffs include the use of disposable cuffs, washing, and wiping with ethanol. Among them, disposable cuffs are expensive but will be more widely used in the future. Washing is also effective in maintaining the cleanliness of cuffs, although it damages their texture. After wiping with alcohol, a cuff cannot be used for about 30 min, but this is a practical method for the maintenance of cleanliness. In the hospital investigated, after the high incidence of MRSA contamination of blood pressure cuffs was clarified, it was decided that they should be wiped with alcohol once daily (cuffs are wiped with alcohol each time after use by patients with MRSA colonization/infection) and washed at 7-day to 1-month intervals. We have seen a decline in MRSA cuff contamination on our wards since we brought in the daily alcohol wipe procedure.
